# Medical News and Bulletin

**Published:** 1843-04-15

**Authors:** 


### UNIVERSITY OF PENNSYLVANIA.

At a public commencement held the 31st day of March, 1843, in the Hall of the Musical Fund Society, Locust street, the following gentlemen received the Degree of Doctor of Medicine, at the hands of the Rev. JOHN LUDLOW, D. D., Provost.

An address was then delivered by Dr. HUGH L. HODGE, Prof. of Obstetrics.

Thomas C. Arrington, North Carolina; Samuel K. Ashton, Philadelphia; Charles L. Baker, Pennsylvania; Charles E. Ballard, North Carolina; William E. Barnes, Pennsylvania; George H. Beaumont, Pennsylvania; Richard F. Berkeley, Virginia; John Milton Best, Kentucky; Edward F. Birckhead, Virginia; John Francis Bird, Philadelphia; Jesse T. Boone, Ohio; William E. Bowling, Kentucky; Samuel Bradley, South Carolina; James C. Brandon, Mississippi; Etheldred E. Briggs, Virginia; Jesse Burgess, Pennsylvania; George H. Burgin, Jr., Philadelphia; George N. Burwell, New York, Ralph Butterfield, Massachusetts; Flavius A. Byrd, Alabama; Alexander H. Campbell, Pennsylvania; Walter Cary, New York; William A. Cheatham, Tennessee; Isaiah D. Clawson, New Jersey; William H. Coleman, Alabama; James H. Conway, Virginia; Thomas B. Cooper, Pennsylvania; Richard H. Coryell, New Jersey; Benjamin W. Curtis, Maine; Charles H. Davis, Virginia; James Dougal, Pennsylvania; Samuel E. Duffield, Pennsylvania; Simeon J. Eddins, Alabama; James L. Elliott, Pennsylvania; Benjamin F. Fessenden, North Carolina; Oswald B. Finney, (M. D.) Virginia; Matthew Gayle, Alabama; James Gower, Tennessee; Fleming T. Grantland, Georgia; Jacob Grigg, Pennsylvania; John L. Hadley, Tennessee; Alfred C. Haines, Ohio; William P. Hall, Virginia; Thomas S. Harper, Pennsylvania; William A. Harris, Philadelphia; Benjamin S. Harrison, North Carolina; Elwood Harvey, Pennsylvania; Rufus S. Harwell, Tennessee; Samuel A. Hinton, Virginia; George W. Holstein, Pennsylvania; Benjamin Housekeeper, Philadelphia; William G. J. Hunter, Tennessee; Wiley Jenkins, Mississippi; Benjamin F. Jones, Alabama; John R. Jones, Pennsylvania; Charles R. Kemper, Virginia; John J. Kerr, Philadelphia; Thomas H. Laird, Virginia; Nazareth Leggett, North Carolina; John C. Lehman, Philadelphia; John B. Lindsley, Tennessee; George H. Lyman, Massachusetts; Robert T. Maccoun, Pennsylvania; Robert L. M'Guire, Virginia; Gabriel S. Martin, Georgia; Thomas W. Mason, Alabama; William Mayburry, Pennsylvania; Thomas S. Mercer, Maryland; John Merritt, Delaware; John T. Metcalfe, Mississippi; Henry C. Miller, South Carolina; George W. Moor, Massachusetts; William O. Morrison, Louisiana; Thomas M. Morton, Kentucky; Robert P. Moss, South Carolina; Robert Murray, Maryland; George J. Musgrave, North Carolina; Clurin V. Nash, Alabama; Nathaniel R. Newkirk, New Jersey; Charles H. Nichols, Rhode Island; Edward H. M. Parham, Virginia; Richmond N. Pearson, North Carolina; Jesse A. Penniman, Massachusetts; Lawrence S. Pepper, Philadelphia; John T. Pope, North Carolina; Henry Race, New Jersey; William N. Raines, Virginia; Thomas Reynolds, North Carolina; William M. L. Rickards, Delaware; John C. Rogers, North Carolina; Fitzwilliam Sargent, Philadelphia; Thomas J. Saunders, New Jersey; John S. Schanck, New Jersey; James K. Shivers, Philadelphia; Edward L. Simmons, Virginia; Spyers Singleton, North Carolina; Henry T. Slemmer, Pennsylvania; Richard H. Stabler, District of Columbia; Nathaniel Stockwell, Mississippi; Magnus W. Stribling, Virginia; Charles H. Taylor, Pennsylvania; Jason B. Thomas, Massachusetts; Francis O. Ticknor, Georgia; George B. Twitchell, New Hampshire; John W. Tyler, Virginia; George L. Upshur, Virginia; Sydenham Walke, Virginia; William Walker, Tennessee; Robert E. Wall, Virginia; Robert Wellford, Virginia; John J. Wilson, Pennsylvania; John R. Wilson, Virginia; William B. Wilson, Pennsylvania; Samuel Wolff, Pennsylvania.

Anderson Bagley, of Virginia, is also entitled to a degree, but from an accidental informality, whereby his name was not in time for the Mandamus of the Board of Trustees, the conferring of it is postponed to July next.

---

At the Commencement held in July, 1842, the Degree of Doctor of Medicine was conferred upon

Benjamin F. Nicholls, South Carolina; Washington G. Nugent, Pennsylvania; Thomas S. Roycroft, New York; Reuben J. Thomas, Virginia.

Total, 118.

W. E. HORNER,
*Dean of Medical Faculty.*

---

### "THE BULLETIN OF MEDICAL SCIENCE."

This is the title of a new medical periodical, issued by Messrs. Haswell & Barrington, and edited by Dr. JOHN BELL. We have received the 1st and 2d Nos. They contain selections from the foreign journals, with Editorials and Bibliography. The abilities of the editor are a safe guaranty for the successful prosecution of the enterprise. The numbers already issued promise well. It is issued monthly.

---

We have received and read with much pleasure Dr. BARTLETT's Valedictory Address to the Graduates of Transylvania University, Lexington. It contains much sound practical advice.

---

Several of the late numbers of the Western Journal of Medicine and Surgery contain an elaborate "Experimental and Critical Inquiry into the Nature and Treatment of Wounds of the Intestines," by Prof. GROSS, of the Louisville Medical Institute. On its completion it will, we suppose, be published separately, and we shall then lay the principal points of interest it contains before our readers.

---

### JOHN HUNTER AS A LECTURER.

Without fluency and vivacity, a lecturer can rarely be popular; and hence John Hunter's lectures were but thinly attended. Like Milton, he was probably content if he could "a fit audience find, though few;" and he might, indeed, have been satisfied could he have anticipated the future glories of his pupils. Let us estimate his lectures, not by a cold analysis, but by their effects. When Demosthenes had thundered forth a Philippic, the Athenians did not say, "What a fine oration!" but, "let us march against Philip."

*Arnott's Hunterian Oration.*

